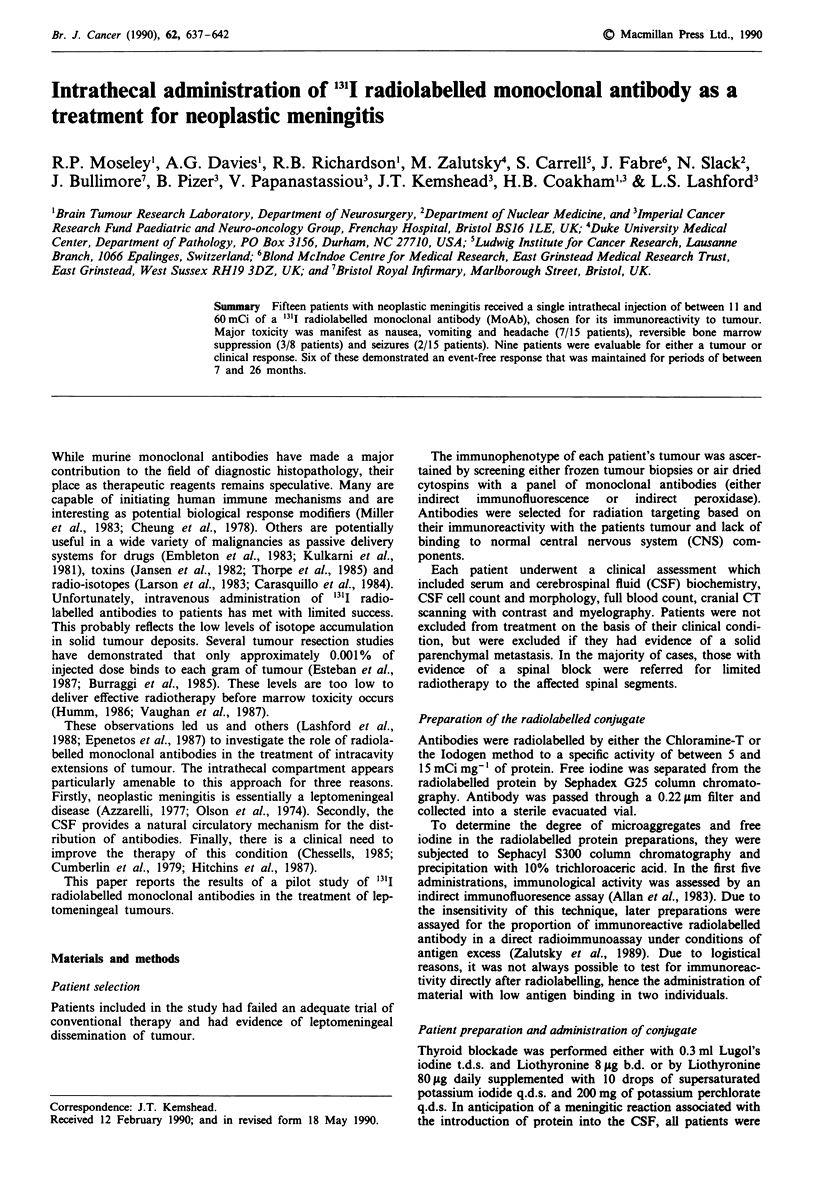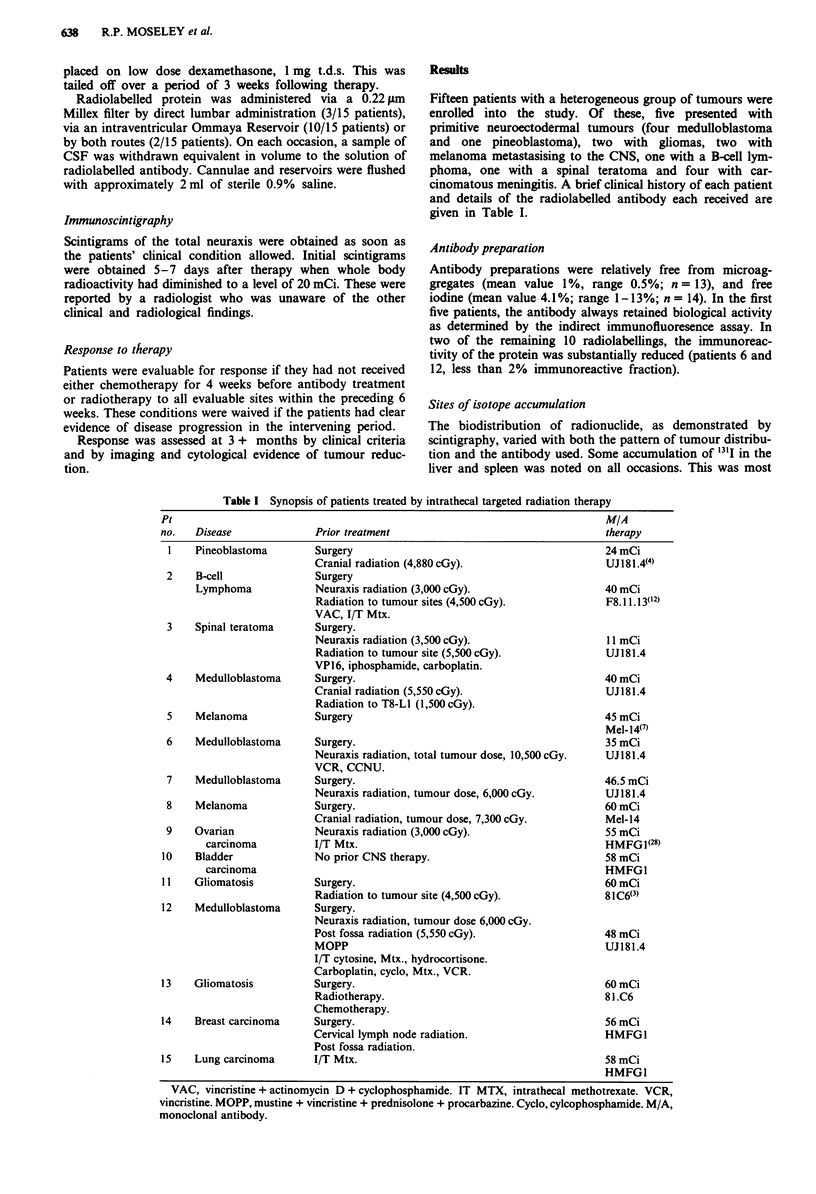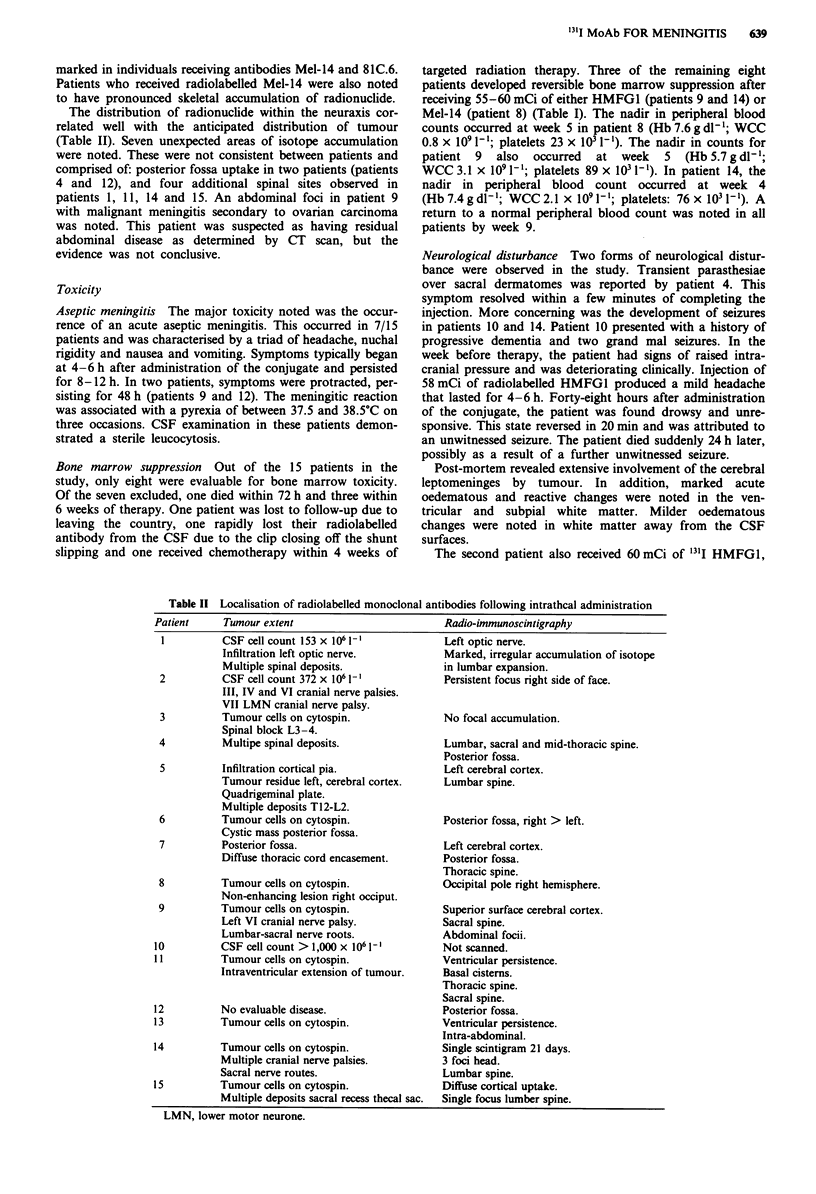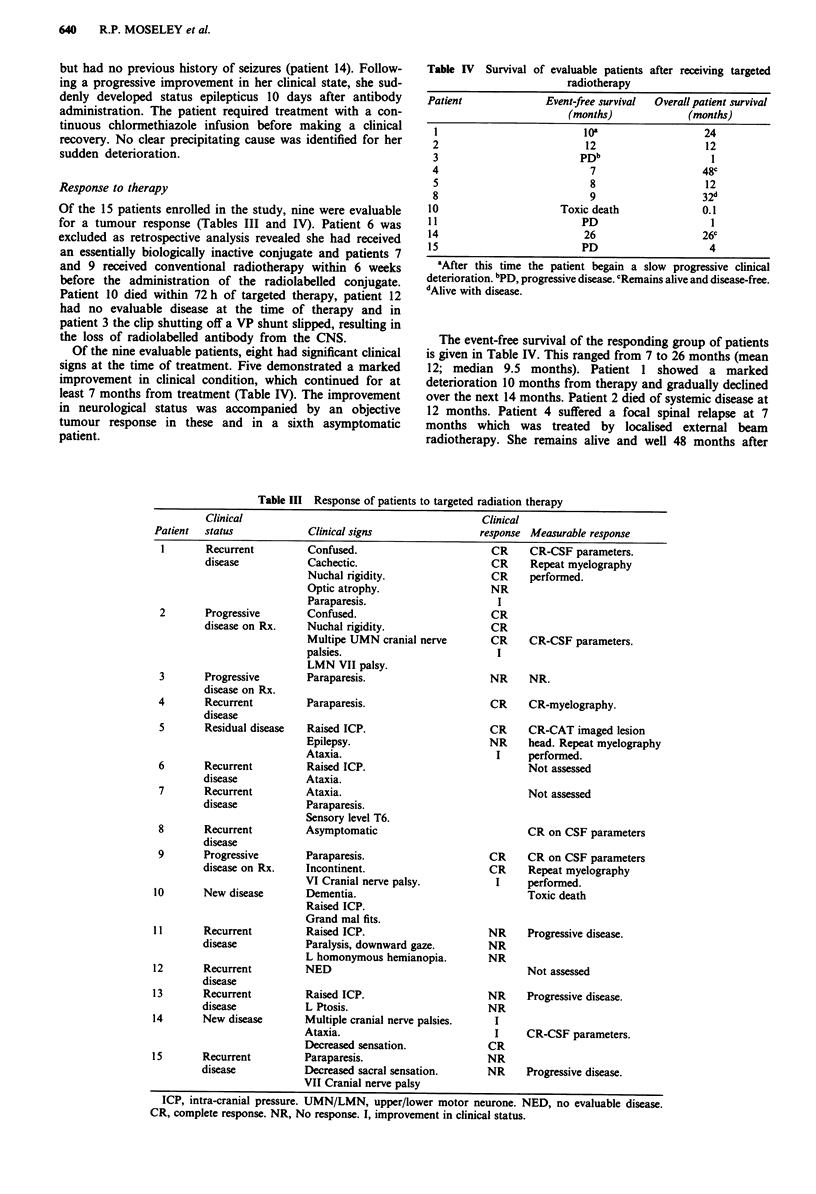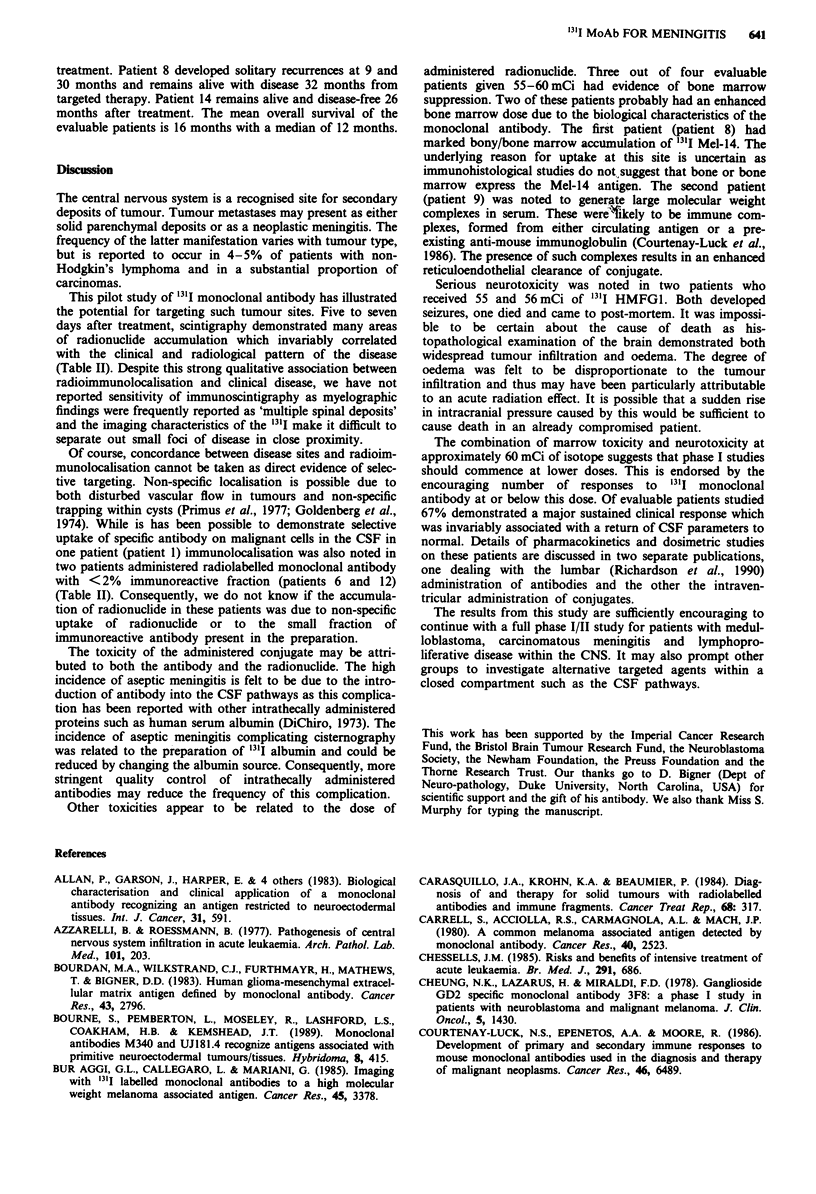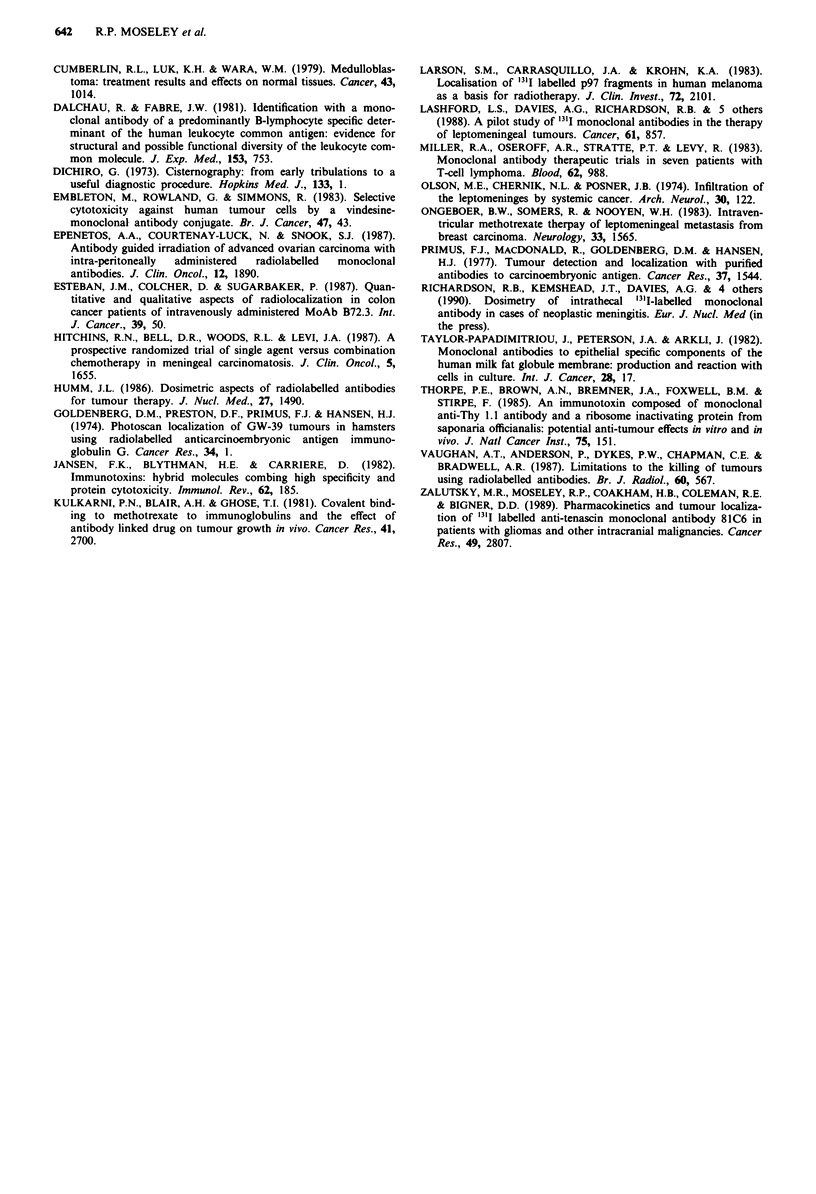# Intrathecal administration of 131I radiolabelled monoclonal antibody as a treatment for neoplastic meningitis.

**DOI:** 10.1038/bjc.1990.345

**Published:** 1990-10

**Authors:** R. P. Moseley, A. G. Davies, R. B. Richardson, M. Zalutsky, S. Carrell, J. Fabre, N. Slack, J. Bullimore, B. Pizer, V. Papanastassiou

**Affiliations:** Department of Neurosurgery, Frenchay Hospital, Bristol, UK.

## Abstract

Fifteen patients with neoplastic meningitis received a single intrathecal injection of between 11 and 60 mCi of a 131I radiolabelled monoclonal antibody (MoAb), chosen for its immunoreactivity to tumour. Major toxicity was manifest as nausea, vomiting and headache (7/15 patients), reversible bone marrow suppression (3/8 patients) and seizures (2/15 patients). Nine patients were evaluable for either a tumour or clinical response. Six of these demonstrated an event-free response that was maintained for periods of between 7 and 26 months.


Br. J. Cancer (1990), 62, 637–642 © Macmillan Press Ltd., 1990

### Intrathecal administration of $^{131}$I radiolabelled monoclonal antibody as a treatment for neoplastic meningitis

R.P. Moseley[1], A.G. Davies[1], R.B. Richardson[1], M. Zalutsky[4], S. Carrell[5], J. Fabre[6], N. Slack[2], J. Bullimore[7], B. Pizer[3], V. Papanastassiou[3], J.T. Kemshead[3], H.B. Coakham[1,3] & L.S. Lashford[3]

[1]*Brain Tumour Research Laboratory, Department of Neurosurgery,* [2]*Department of Nuclear Medicine, and* [3]*Imperial Cancer Research Fund Paediatric and Neuro-oncology Group, Frenchay Hospital, Bristol BS16 1LE, UK;* [4]*Duke University Medical Center, Department of Pathology, PO Box 3156, Durham, NC 27710, USA;* [5]*Ludwig Institute for Cancer Research, Lausanne Branch, 1066 Epalinges, Switzerland;* [6]*Blond McIndoe Centre for Medical Research, East Grinstead Medical Research Trust, East Grinstead, West Sussex RH19 3DZ, UK; and* [7]*Bristol Royal Infirmary, Marlborough Street, Bristol, UK.*

**Summary** Fifteen patients with neoplastic meningitis received a single intrathecal injection of between 11 and 60 mCi of a $^{131}$I radiolabelled monoclonal antibody (MoAb), chosen for its immunoreactivity to tumour. Major toxicity was manifest as nausea, vomiting and headache (7/15 patients), reversible bone marrow suppression (3/8 patients) and seizures (2/15 patients). Nine patients were evaluable for either a tumour or clinical response. Six of these demonstrated an event-free response that was maintained for periods of between 7 and 26 months.

While murine monoclonal antibodies have made a major contribution to the field of diagnostic histopathology, their place as therapeutic reagents remains speculative. Many are capable of initiating human immune mechanisms and are interesting as potential biological response modifiers (Miller *et al.*, 1983; Cheung *et al.*, 1978). Others are potentially useful in a wide variety of malignancies as passive delivery systems for drugs (Embleton *et al.*, 1983; Kulkarni *et al.*, 1981), toxins (Jansen *et al.*, 1982; Thorpe *et al.*, 1985) and radio-isotopes (Larson *et al.*, 1983; Carasquillo *et al.*, 1984). Unfortunately, intravenous administration of $^{131}$I radiolabelled antibodies to patients has met with limited success. This probably reflects the low levels of isotope accumulation in solid tumour deposits. Several tumour resection studies have demonstrated that only approximately 0.001% of injected dose binds to each gram of tumour (Esteban *et al.*, 1987; Burraggi *et al.*, 1985). These levels are too low to deliver effective radiotherapy before marrow toxicity occurs (Humm, 1986; Vaughan *et al.*, 1987).

These observations led us and others (Lashford *et al.*, 1988; Epenetos *et al.*, 1987) to investigate the role of radiolabelled monoclonal antibodies in the treatment of intracavity extensions of tumour. The intrathecal compartment appears particularly amenable to this approach for three reasons. Firstly, neoplastic meningitis is essentially a leptomeningeal disease (Azzarelli, 1977; Olson *et al.*, 1974). Secondly, the CSF provides a natural circulatory mechanism for the distribution of antibodies. Finally, there is a clinical need to improve the therapy of this condition (Chessells, 1985; Cumberlin *et al.*, 1979; Hitchins *et al.*, 1987).

This paper reports the results of a pilot study of $^{131}$I radiolabelled monoclonal antibodies in the treatment of leptomeningeal tumours.

### Materials and methods

### *Patient selection*

Patients included in the study had failed an adequate trial of conventional therapy and had evidence of leptomeningeal dissemination of tumour.

The immunophenotype of each patient's tumour was ascertained by screening either frozen tumour biopsies or air dried cytospins with a panel of monoclonal antibodies (either indirect immunofluorescence or indirect peroxidase). Antibodies were selected for radiation targeting based on their immunoreactivity with the patients tumour and lack of binding to normal central nervous system (CNS) components.

Each patient underwent a clinical assessment which included serum and cerebrospinal fluid (CSF) biochemistry, CSF cell count and morphology, full blood count, cranial CT scanning with contrast and myelography. Patients were not excluded from treatment on the basis of their clinical condition, but were excluded if they had evidence of a solid parenchymal metastasis. In the majority of cases, those with evidence of a spinal block were referred for limited radiotherapy to the affected spinal segments.

### *Preparation of the radiolabelled conjugate*

Antibodies were radiolabelled by either the Chloramine-T or the Iodogen method to a specific activity of between 5 and 15 mCi mg$^{-1}$ of protein. Free iodine was separated from the radiolabelled protein by Sephadex G25 column chromatography. Antibody was passed through a 0.22 $\mu$m filter and collected into a sterile evacuated vial.

To determine the degree of microaggregates and free iodine in the radiolabelled protein preparations, they were subjected to Sephacyl S300 column chromatography and precipitation with 10% trichloroaceric acid. In the first five administrations, immunological activity was assessed by an indirect immunofluoresence assay (Allan *et al.*, 1983). Due to the insensitivity of this technique, later preparations were assayed for the proportion of immunoreactive radiolabelled antibody in a direct radioimmunoassay under conditions of antigen excess (Zalutsky *et al.*, 1989). Due to logistical reasons, it was not always possible to test for immunoreactivity directly after radiolabelling, hence the administration of material with low antigen binding in two individuals.

### *Patient preparation and administration of conjugate*

Thyroid blockade was performed either with 0.3 ml Lugol's iodine t.d.s. and Liothyronine 8 $\mu$g b.d. or by Liothyronine 80 $\mu$g daily supplemented with 10 drops of supersaturated potassium iodide q.d.s. and 200 mg of potassium perchlorate q.d.s. In anticipation of a meningitic reaction associated with the introduction of protein into the CSF, all patients were

Correspondence: J.T. Kemshead.
Received 12 February 1990; and in revised form 18 May 1990.

placed on low dose dexamethasone, 1 mg t.d.s. This was tailed off over a period of 3 weeks following therapy.

Radiolabelled protein was administered via a 0.22 µm Millex filter by direct lumbar administration (3/15 patients), via an intraventricular Ommaya Reservoir (10/15 patients) or by both routes (2/15 patients). On each occasion, a sample of CSF was withdrawn equivalent in volume to the solution of radiolabelled antibody. Cannulae and reservoirs were flushed with approximately 2 ml of sterile 0.9% saline.

*Immunoscintigraphy*

Scintigrams of the total neuraxis were obtained as soon as the patients' clinical condition allowed. Initial scintigrams were obtained 5–7 days after therapy when whole body radioactivity had diminished to a level of 20 mCi. These were reported by a radiologist who was unaware of the other clinical and radiological findings.

*Response to therapy*

Patients were evaluable for response if they had not received either chemotherapy for 4 weeks before antibody treatment or radiotherapy to all evaluable sites within the preceding 6 weeks. These conditions were waived if the patients had clear evidence of disease progression in the intervening period.

Response was assessed at 3+ months by clinical criteria and by imaging and cytological evidence of tumour reduction.

### Results

Fifteen patients with a heterogeneous group of tumours were enrolled into the study. Of these, five presented with primitive neuroectodermal tumours (four medulloblastoma and one pineoblastoma), two with gliomas, two with melanoma metastasising to the CNS, one with a B-cell lymphoma, one with a spinal teratoma and four with carcinomatous meningitis. A brief clinical history of each patient and details of the radiolabelled antibody each received are given in Table I.

*Antibody preparation*

Antibody preparations were relatively free from microaggregates (mean value 1%, range 0.5%; $n = 13$), and free iodine (mean value 4.1%; range 1–13%; $n = 14$). In the first five patients, the antibody always retained biological activity as determined by the indirect immunofluoresence assay. In two of the remaining 10 radiolabellings, the immunoreactivity of the protein was substantially reduced (patients 6 and 12, less than 2% immunoreactive fraction).

*Sites of isotope accumulation*

The biodistribution of radionuclide, as demonstrated by scintigraphy, varied with both the pattern of tumour distribution and the antibody used. Some accumulation of $^{131}I$ in the liver and spleen was noted on all occasions. This was most

**Table I** Synopsis of patients treated by intrathecal targeted radiation therapy

| *Pt no.* | *Disease* | *Prior treatment* | *M/A therapy* |
|---|---|---|---|
| 1 | Pineoblastoma | Surgery<br>Cranial radiation (4,880 cGy). | 24 mCi<br>UJ181.4[4] |
| 2 | B-cell Lymphoma | Surgery<br>Neuraxis radiation (3,000 cGy).<br>Radiation to tumour sites (4,500 cGy).<br>VAC, I/T Mtx. | 40 mCi<br>F8.11.13[12] |
| 3 | Spinal teratoma | Surgery.<br>Neuraxis radiation (3,500 cGy).<br>Radiation to tumour site (5,500 cGy).<br>VP16, iphosphamide, carboplatin. | 11 mCi<br>UJ181.4 |
| 4 | Medulloblastoma | Surgery.<br>Cranial radiation (5,550 cGy).<br>Radiation to T8-L1 (1,500 cGy). | 40 mCi<br>UJ181.4 |
| 5 | Melanoma | Surgery | 45 mCi<br>Mel-14[7] |
| 6 | Medulloblastoma | Surgery.<br>Neuraxis radiation, total tumour dose, 10,500 cGy.<br>VCR, CCNU. | 35 mCi<br>UJ181.4 |
| 7 | Medulloblastoma | Surgery.<br>Neuraxis radiation, tumour dose, 6,000 cGy. | 46.5 mCi<br>UJ181.4 |
| 8 | Melanoma | Surgery.<br>Cranial radiation, tumour dose, 7,300 cGy. | 60 mCi<br>Mel-14 |
| 9 | Ovarian carcinoma | Neuraxis radiation (3,000 cGy).<br>I/T Mtx. | 55 mCi<br>HMFG1[28] |
| 10 | Bladder carcinoma | No prior CNS therapy. | 58 mCi<br>HMFG1 |
| 11 | Gliomatosis | Surgery.<br>Radiation to tumour site (4,500 cGy). | 60 mCi<br>81C6[3] |
| 12 | Medulloblastoma | Surgery.<br>Neuraxis radiation, tumour dose 6,000 cGy.<br>Post fossa radiation (5,550 cGy).<br>MOPP<br>I/T cytosine, Mtx., hydrocortisone.<br>Carboplatin, cyclo, Mtx., VCR. | 48 mCi<br>UJ181.4 |
| 13 | Gliomatosis | Surgery.<br>Radiotherapy.<br>Chemotherapy. | 60 mCi<br>81.C6 |
| 14 | Breast carcinoma | Surgery.<br>Cervical lymph node radiation.<br>Post fossa radiation. | 56 mCi<br>HMFG1 |
| 15 | Lung carcinoma | I/T Mtx. | 58 mCi<br>HMFG1 |

VAC, vincristine + actinomycin D + cyclophosphamide. IT MTX, intrathecal methotrexate. VCR, vincristine. MOPP, mustine + vincristine + prednisolone + procarbazine. Cyclo, cylcophosphamide. M/A, monoclonal antibody.

marked in individuals receiving antibodies Mel-14 and 81C.6. Patients who received radiolabelled Mel-14 were also noted to have pronounced skeletal accumulation of radionuclide.

The distribution of radionuclide within the neuraxis correlated well with the anticipated distribution of tumour (Table II). Seven unexpected areas of isotope accumulation were noted. These were not consistent between patients and comprised of: posterior fossa uptake in two patients (patients 4 and 12), and four additional spinal sites observed in patients 1, 11, 14 and 15. An abdominal foci in patient 9 with malignant meningitis secondary to ovarian carcinoma was noted. This patient was suspected as having residual abdominal disease as determined by CT scan, but the evidence was not conclusive.

### *Toxicity*

*Aseptic meningitis* The major toxicity noted was the occurrence of an acute aseptic meningitis. This occurred in 7/15 patients and was characterised by a triad of headache, nuchal rigidity and nausea and vomiting. Symptoms typically began at 4–6 h after administration of the conjugate and persisted for 8–12 h. In two patients, symptoms were protracted, persisting for 48 h (patients 9 and 12). The meningitic reaction was associated with a pyrexia of between 37.5 and 38.5°C on three occasions. CSF examination in these patients demonstrated a sterile leucocytosis.

*Bone marrow suppression* Out of the 15 patients in the study, only eight were evaluable for bone marrow toxicity. Of the seven excluded, one died within 72 h and three within 6 weeks of therapy. One patient was lost to follow-up due to leaving the country, one rapidly lost their radiolabelled antibody from the CSF due to the clip closing off the shunt slipping and one received chemotherapy within 4 weeks of targeted radiation therapy. Three of the remaining eight patients developed reversible bone marrow suppression after receiving 55–60 mCi of either HMFG1 (patients 9 and 14) or Mel-14 (patient 8) (Table I). The nadir in peripheral blood counts occurred at week 5 in patient 8 (Hb 7.6 g $dl^{-1}$; WCC $0.8 \times 10^9 l^{-1}$; platelets $23 \times 10^3 l^{-1}$). The nadir in counts for patient 9 also occurred at week 5 (Hb 5.7 g $dl^{-1}$; WCC $3.1 \times 10^9 l^{-1}$; platelets $89 \times 10^3 l^{-1}$). In patient 14, the nadir in peripheral blood count occurred at week 4 (Hb 7.4 g $dl^{-1}$; WCC $2.1 \times 10^9 l^{-1}$; platelets: $76 \times 10^3 l^{-1}$). A return to a normal peripheral blood count was noted in all patients by week 9.

*Neurological disturbance* Two forms of neurological disturbance were observed in the study. Transient parasthesiae over sacral dermatomes was reported by patient 4. This symptom resolved within a few minutes of completing the injection. More concerning was the development of seizures in patients 10 and 14. Patient 10 presented with a history of progressive dementia and two grand mal seizures. In the week before therapy, the patient had signs of raised intracranial pressure and was deteriorating clinically. Injection of 58 mCi of radiolabelled HMFG1 produced a mild headache that lasted for 4–6 h. Forty-eight hours after administration of the conjugate, the patient was found drowsy and unresponsive. This state reversed in 20 min and was attributed to an unwitnessed seizure. The patient died suddenly 24 h later, possibly as a result of a further unwitnessed seizure.

Post-mortem revealed extensive involvement of the cerebral leptomeninges by tumour. In addition, marked acute oedematous and reactive changes were noted in the ventricular and subpial white matter. Milder oedematous changes were noted in white matter away from the CSF surfaces.

The second patient also received 60 mCi of $^{131}$I HMFG1,

**Table II** Localisation of radiolabelled monoclonal antibodies following intrathcal administration

| *Patient* | *Tumour extent* | *Radio-immunoscintigraphy* |
|---|---|---|
| 1 | CSF cell count $153 \times 10^6 l^{-1}$<br>Infiltration left optic nerve.<br>Multiple spinal deposits. | Left optic nerve.<br>Marked, irregular accumulation of isotope in lumbar expansion. |
| 2 | CSF cell count $372 \times 10^6 l^{-1}$<br>III, IV and VI cranial nerve palsies.<br>VII LMN cranial nerve palsy. | Persistent focus right side of face. |
| 3 | Tumour cells on cytospin.<br>Spinal block L3–4. | No focal accumulation. |
| 4 | Multipe spinal deposits. | Lumbar, sacral and mid-thoracic spine.<br>Posterior fossa. |
| 5 | Infiltration cortical pia.<br>Tumour residue left, cerebral cortex.<br>Quadrigeminal plate.<br>Multiple deposits T12-L2. | Left cerebral cortex.<br>Lumbar spine. |
| 6 | Tumour cells on cytospin.<br>Cystic mass posterior fossa. | Posterior fossa, right > left. |
| 7 | Posterior fossa.<br>Diffuse thoracic cord encasement. | Left cerebral cortex.<br>Posterior fossa.<br>Thoracic spine. |
| 8 | Tumour cells on cytospin.<br>Non-enhancing lesion right occiput. | Occipital pole right hemisphere. |
| 9 | Tumour cells on cytospin.<br>Left VI cranial nerve palsy.<br>Lumbar-sacral nerve roots. | Superior surface cerebral cortex.<br>Sacral spine.<br>Abdominal focii. |
| 10 | CSF cell count $> 1{,}000 \times 10^6 l^{-1}$ | Not scanned. |
| 11 | Tumour cells on cytospin.<br>Intraventricular extension of tumour. | Ventricular persistence.<br>Basal cisterns.<br>Thoracic spine.<br>Sacral spine. |
| 12 | No evaluable disease. | Posterior fossa. |
| 13 | Tumour cells on cytospin. | Ventricular persistence.<br>Intra-abdominal. |
| 14 | Tumour cells on cytospin.<br>Multiple cranial nerve palsies.<br>Sacral nerve routes. | Single scintigram 21 days.<br>3 foci head.<br>Lumbar spine. |
| 15 | Tumour cells on cytospin.<br>Multiple deposits sacral recess thecal sac. | Diffuse cortical uptake.<br>Single focus lumber spine. |

LMN, lower motor neurone.

but had no previous history of seizures (patient 14). Following a progressive improvement in her clinical state, she suddenly developed status epilepticus 10 days after antibody administration. The patient required treatment with a continuous chlormethiazole infusion before making a clinical recovery. No clear precipitating cause was identified for her sudden deterioration.

*Response to therapy*

Of the 15 patients enrolled in the study, nine were evaluable for a tumour response (Tables III and IV). Patient 6 was excluded as retrospective analysis revealed she had received an essentially biologically inactive conjugate and patients 7 and 9 received conventional radiotherapy within 6 weeks before the administration of the radiolabelled conjugate. Patient 10 died within 72 h of targeted therapy, patient 12 had no evaluable disease at the time of therapy and in patient 3 the clip shutting off a VP shunt slipped, resulting in the loss of radiolabelled antibody from the CNS.

Of the nine evaluable patients, eight had significant clinical signs at the time of treatment. Five demonstrated a marked improvement in clinical condition, which continued for at least 7 months from treatment (Table IV). The improvement in neurological status was accompanied by an objective tumour response in these and in a sixth asymptomatic patient.

**Table IV** Survival of evaluable patients after receiving targeted radiotherapy

| *Patient* | *Event-free survival (months)* | *Overall patient survival (months)* |
|---|---|---|
| 1 | 10[a] | 24 |
| 2 | 12 | 12 |
| 3 | PD[b] | 1 |
| 4 | 7 | 48[c] |
| 5 | 8 | 12 |
| 8 | 9 | 32[d] |
| 10 | Toxic death | 0.1 |
| 11 | PD | 1 |
| 14 | 26 | 26[c] |
| 15 | PD | 4 |

[a]After this time the patient begain a slow progressive clinical deterioration. [b]PD, progressive disease. [c]Remains alive and disease-free. [d]Alive with disease.

The event-free survival of the responding group of patients is given in Table IV. This ranged from 7 to 26 months (mean 12; median 9.5 months). Patient 1 showed a marked deterioration 10 months from therapy and gradually declined over the next 14 months. Patient 2 died of systemic disease at 12 months. Patient 4 suffered a focal spinal relapse at 7 months which was treated by localised external beam radiotherapy. She remains alive and well 48 months after

**Table III** Response of patients to targeted radiation therapy

| *Patient* | *Clinical status* | *Clinical signs* | *Clinical response* | *Measurable response* |
|---|---|---|---|---|
| 1 | Recurrent disease | Confused.<br>Cachectic.<br>Nuchal rigidity.<br>Optic atrophy.<br>Paraparesis. | CR<br>CR<br>CR<br>NR<br>I | CR-CSF parameters. Repeat myelography performed. |
| 2 | Progressive disease on Rx. | Confused.<br>Nuchal rigidity.<br>Multipe UMN cranial nerve palsies.<br>LMN VII palsy. | CR<br>CR<br>CR<br>I | CR-CSF parameters. |
| 3 | Progressive disease on Rx. | Paraparesis. | NR | NR. |
| 4 | Recurrent disease | Paraparesis. | CR | CR-myelography. |
| 5 | Residual disease | Raised ICP.<br>Epilepsy.<br>Ataxia. | CR<br>NR<br>I | CR-CAT imaged lesion head. Repeat myelography performed. |
| 6 | Recurrent disease | Raised ICP.<br>Ataxia. | | Not assessed |
| 7 | Recurrent disease | Ataxia.<br>Paraparesis.<br>Sensory level T6. | | Not assessed |
| 8 | Recurrent disease | Asymptomatic | | CR on CSF parameters |
| 9 | Progressive disease on Rx. | Paraparesis.<br>Incontinent.<br>VI Cranial nerve palsy. | CR<br>CR<br>I | CR on CSF parameters Repeat myelography performed. |
| 10 | New disease | Dementia.<br>Raised ICP.<br>Grand mal fits. | | Toxic death |
| 11 | Recurrent disease | Raised ICP.<br>Paralysis, downward gaze.<br>L homonymous hemianopia. | NR<br>NR<br>NR | Progressive disease. |
| 12 | Recurrent disease | NED | | Not assessed |
| 13 | Recurrent disease | Raised ICP.<br>L Ptosis. | NR<br>NR | Progressive disease. |
| 14 | New disease | Multiple cranial nerve palsies.<br>Ataxia.<br>Decreased sensation. | I<br>I<br>CR | CR-CSF parameters. |
| 15 | Recurrent disease | Paraparesis.<br>Decreased sacral sensation.<br>VII Cranial nerve palsy | NR<br>NR | Progressive disease. |

ICP, intra-cranial pressure. UMN/LMN, upper/lower motor neurone. NED, no evaluable disease. CR, complete response. NR, No response. I, improvement in clinical status.

treatment. Patient 8 developed solitary recurrences at 9 and 30 months and remains alive with disease 32 months from targeted therapy. Patient 14 remains alive and disease-free 26 months after treatment. The mean overall survival of the evaluable patients is 16 months with a median of 12 months.

### Discussion

The central nervous system is a recognised site for secondary deposits of tumour. Tumour metastases may present as either solid parenchymal deposits or as a neoplastic meningitis. The frequency of the latter manifestation varies with tumour type, but is reported to occur in 4–5% of patients with non-Hodgkin's lymphoma and in a substantial proportion of carcinomas.

This pilot study of $^{131}$I monoclonal antibody has illustrated the potential for targeting such tumour sites. Five to seven days after treatment, scintigraphy demonstrated many areas of radionuclide accumulation which invariably correlated with the clinical and radiological pattern of the disease (Table II). Despite this strong qualitative association between radioimmunolocalisation and clinical disease, we have not reported sensitivity of immunoscintigraphy as myelographic findings were frequently reported as 'multiple spinal deposits' and the imaging characteristics of the $^{131}$I make it difficult to separate out small foci of disease in close proximity.

Of course, concordance between disease sites and radioimmunolocalisation cannot be taken as direct evidence of selective targeting. Non-specific localisation is possible due to both disturbed vascular flow in tumours and non-specific trapping within cysts (Primus *et al.*, 1977; Goldenberg *et al.*, 1974). While is has been possible to demonstrate selective uptake of specific antibody on malignant cells in the CSF in one patient (patient 1) immunolocalisation was also noted in two patients administered radiolabelled monoclonal antibody with $<2\%$ immunoreactive fraction (patients 6 and 12) (Table II). Consequently, we do not know if the accumulation of radionuclide in these patients was due to non-specific uptake of radionuclide or to the small fraction of immunoreactive antibody present in the preparation.

The toxicity of the administered conjugate may be attributed to both the antibody and the radionuclide. The high incidence of aseptic meningitis is felt to be due to the introduction of antibody into the CSF pathways as this complication has been reported with other intrathecally administered proteins such as human serum albumin (DiChiro, 1973). The incidence of aseptic meningitis complicating cisternography was related to the preparation of $^{131}$I albumin and could be reduced by changing the albumin source. Consequently, more stringent quality control of intrathecally administered antibodies may reduce the frequency of this complication.

Other toxicities appear to be related to the dose of administered radionuclide. Three out of four evaluable patients given 55–60 mCi had evidence of bone marrow suppression. Two of these patients probably had an enhanced bone marrow dose due to the biological characteristics of the monoclonal antibody. The first patient (patient 8) had marked bony/bone marrow accumulation of $^{131}$I Mel-14. The underlying reason for uptake at this site is uncertain as immunohistological studies do not suggest that bone or bone marrow express the Mel-14 antigen. The second patient (patient 9) was noted to generate large molecular weight complexes in serum. These were likely to be immune complexes, formed from either circulating antigen or a pre-existing anti-mouse immunoglobulin (Courtenay-Luck *et al.*, 1986). The presence of such complexes results in an enhanced reticuloendothelial clearance of conjugate.

Serious neurotoxicity was noted in two patients who received 55 and 56 mCi of $^{131}$I HMFG1. Both developed seizures, one died and came to post-mortem. It was impossible to be certain about the cause of death as histopathological examination of the brain demonstrated both widespread tumour infiltration and oedema. The degree of oedema was felt to be disproportionate to the tumour infiltration and thus may have been particularly attributable to an acute radiation effect. It is possible that a sudden rise in intracranial pressure caused by this would be sufficient to cause death in an already compromised patient.

The combination of marrow toxicity and neurotoxicity at approximately 60 mCi of isotope suggests that phase I studies should commence at lower doses. This is endorsed by the encouraging number of responses to $^{131}$I monoclonal antibody at or below this dose. Of evaluable patients studied 67% demonstrated a major sustained clinical response which was invariably associated with a return of CSF parameters to normal. Details of pharmacokinetics and dosimetric studies on these patients are discussed in two separate publications, one dealing with the lumbar (Richardson *et al.*, 1990) administration of antibodies and the other the intraventricular administration of conjugates.

The results from this study are sufficiently encouraging to continue with a full phase I/II study for patients with medulloblastoma, carcinomatous meningitis and lymphoproliferative disease within the CNS. It may also prompt other groups to investigate alternative targeted agents within a closed compartment such as the CSF pathways.

This work has been supported by the Imperial Cancer Research Fund, the Bristol Brain Tumour Research Fund, the Neuroblastoma Society, the Newham Foundation, the Preuss Foundation and the Thorne Research Trust. Our thanks go to D. Bigner (Dept of Neuro-pathology, Duke University, North Carolina, USA) for scientific support and the gift of his antibody. We also thank Miss S. Murphy for typing the manuscript.

### References

ALLAN, P., GARSON, J., HARPER, E. & 4 others (1983). Biological characterisation and clinical application of a monoclonal antibody recognizing an antigen restricted to neuroectodermal tissues. *Int. J. Cancer*, **31**, 591.

AZZARELLI, B. & ROESSMANN, B. (1977). Pathogenesis of central nervous system infiltration in acute leukaemia. *Arch. Pathol. Lab. Med.*, **101**, 203.

BOURDAN, M.A., WILKSTRAND, C.J., FURTHMAYR, H., MATHEWS, T. & BIGNER, D.D. (1983). Human glioma-mesenchymal extracellular matrix antigen defined by monoclonal antibody. *Cancer Res.*, **43**, 2796.

BOURNE, S., PEMBERTON, L., MOSELEY, R., LASHFORD, L.S., COAKHAM, H.B. & KEMSHEAD, J.T. (1989). Monoclonal antibodies M340 and UJ181.4 recognize antigens associated with primitive neuroectodermal tumours/tissues. *Hybridoma*, **8**, 415.

BUR AGGI, G.L., CALLEGARO, L. & MARIANI, G. (1985). Imaging with $^{131}$I labelled monoclonal antibodies to a high molecular weight melanoma associated antigen. *Cancer Res.*, **45**, 3378.

CARASQUILLO, J.A., KROHN, K.A. & BEAUMIER, P. (1984). Diagnosis of and therapy for solid tumours with radiolabelled antibodies and immune fragments. *Cancer Treat Rep.*, **68**: 317.

CARRELL, S., ACCIOLLA, R.S., CARMAGNOLA, A.L. & MACH, J.P. (1980). A common melanoma associated antigen detected by monoclonal antibody. *Cancer Res.*, **40**, 2523.

CHESSELLS, J.M. (1985). Risks and benefits of intensive treatment of acute leukaemia. *Br. Med. J.*, **291**, 686.

CHEUNG, N.K., LAZARUS, H. & MIRALDI, F.D. (1978). Ganglioside GD2 specific monoclonal antibody 3F8: a phase I study in patients with neuroblastoma and malignant melanoma. *J. Clin. Oncol.*, **5**, 1430.

COURTENAY-LUCK, N.S., EPENETOS, A.A. & MOORE, R. (1986). Development of primary and secondary immune responses to mouse monoclonal antibodies used in the diagnosis and therapy of malignant neoplasms. *Cancer Res.*, **46**, 6489.

CUMBERLIN, R.L., LUK, K.H. & WARA, W.M. (1979). Medulloblastoma: treatment results and effects on normal tissues. *Cancer*, **43**, 1014.

DALCHAU, R. & FABRE, J.W. (1981). Identification with a monoclonal antibody of a predominantly B-lymphocyte specific determinant of the human leukocyte common antigen: evidence for structural and possible functional diversity of the leukocyte common molecule. *J. Exp. Med.*, **153**, 753.

DICHIRO, G. (1973). Cisternography: from early tribulations to a useful diagnostic procedure. *Hopkins Med. J.*, **133**, 1.

EMBLETON, M., ROWLAND, G. & SIMMONS, R. (1983). Selective cytotoxicity against human tumour cells by a vindesine-monoclonal antibody conjugate. *Br. J. Cancer*, **47**, 43.

EPENETOS, A.A., COURTENAY-LUCK, N. & SNOOK, S.J. (1987). Antibody guided irradiation of advanced ovarian carcinoma with intra-peritoneally administered radiolabelled monoclonal antibodies. *J. Clin. Oncol.*, **12**, 1890.

ESTEBAN, J.M., COLCHER, D. & SUGARBAKER, P. (1987). Quantitative and qualitative aspects of radiolocalization in colon cancer patients of intravenously administered MoAb B72.3. *Int. J. Cancer.*, **39**, 50.

HITCHINS, R.N., BELL, D.R., WOODS, R.L. & LEVI, J.A. (1987). A prospective randomized trial of single agent versus combination chemotherapy in meningeal carcinomatosis. *J. Clin. Oncol.*, **5**, 1655.

HUMM, J.L. (1986). Dosimetric aspects of radiolabelled antibodies for tumour therapy. *J. Nucl. Med.*, **27**, 1490.

GOLDENBERG, D.M., PRESTON, D.F., PRIMUS, F.J. & HANSEN, H.J. (1974). Photoscan localization of GW-39 tumours in hamsters using radiolabelled anticarcinoembryonic antigen immunoglobulin G. *Cancer Res.*, **34**, 1.

JANSEN, F.K., BLYTHMAN, H.E. & CARRIERE, D. (1982). Immunotoxins: hybrid molecules combing high specificity and protein cytotoxicity. *Immunol. Rev.*, **62**, 185.

KULKARNI, P.N., BLAIR, A.H. & GHOSE, T.I. (1981). Covalent binding to methotrexate to immunoglobulins and the effect of antibody linked drug on tumour growth *in vivo*. *Cancer Res.*, **41**, 2700.

LARSON, S.M., CARRASQUILLO, J.A. & KROHN, K.A. (1983). Localisation of $^{131}$I labelled p97 fragments in human melanoma as a basis for radiotherapy. *J. Clin. Invest.*, **72**, 2101.

LASHFORD, L.S., DAVIES, A.G., RICHARDSON, R.B. & 5 others (1988). A pilot study of $^{131}$I monoclonal antibodies in the therapy of leptomeningeal tumours. *Cancer*, **61**, 857.

MILLER, R.A., OSEROFF, A.R., STRATTE, P.T. & LEVY, R. (1983). Monoclonal antibody therapeutic trials in seven patients with T-cell lymphoma. *Blood*, **62**, 988.

OLSON, M.E., CHERNIK, N.L. & POSNER, J.B. (1974). Infiltration of the leptomeninges by systemic cancer. *Arch. Neurol.*, **30**, 122.

ONGEBOER, B.W., SOMERS, R. & NOOYEN, W.H. (1983). Intraventricular methotrexate therpay of leptomeningeal metastasis from breast carcinoma. *Neurology*, **33**, 1565.

PRIMUS, F.J., MACDONALD, R., GOLDENBERG, D.M. & HANSEN, H.J. (1977). Tumour detection and localization with purified antibodies to carcinoembryonic antigen. *Cancer Res.*, **37**, 1544.

RICHARDSON, R.B., KEMSHEAD, J.T., DAVIES, A.G. & 4 others (1990). Dosimetry of intrathecal $^{131}$I-labelled monoclonal antibody in cases of neoplastic meningitis. *Eur. J. Nucl. Med* (in the press).

TAYLOR-PAPADIMITRIOU, J., PETERSON, J.A. & ARKLI, J. (1982). Monoclonal antibodies to epithelial specific components of the human milk fat globule membrane: production and reaction with cells in culture. *Int. J. Cancer*, **28**, 17.

THORPE, P.E., BROWN, A.N., BREMNER, J.A., FOXWELL, B.M. & STIRPE, F. (1985). An immunotoxin composed of monoclonal anti-Thy 1.1 antibody and a ribosome inactivating protein from saponaria officianalis: potential anti-tumour effects *in vitro* and *in vivo*. *J. Natl Cancer Inst.*, **75**, 151.

VAUGHAN, A.T., ANDERSON, P., DYKES, P.W., CHAPMAN, C.E. & BRADWELL, A.R. (1987). Limitations to the killing of tumours using radiolabelled antibodies. *Br. J. Radiol.*, **60**, 567.

ZALUTSKY, M.R., MOSELEY, R.P., COAKHAM, H.B., COLEMAN, R.E. & BIGNER, D.D. (1989). Pharmacokinetics and tumour localization of $^{131}$I labelled anti-tenascin monoclonal antibody 81C6 in patients with gliomas and other intracranial malignancies. *Cancer Res.*, **49**, 2807.